# Evaluation of a Combined HIV and Geriatrics Clinic for Older People Living with HIV: The Silver Clinic in Brighton, UK

**DOI:** 10.3390/geriatrics5040081

**Published:** 2020-10-19

**Authors:** Tom Levett, Katie Alford, Jonathan Roberts, Zoe Adler, Juliet Wright, Jaime H. Vera

**Affiliations:** 1 Division of Medicine, Brighton and Sussex University Hospitals NHS Trust, UK; t.levett@nhs.net (T.L.); jonathan.roberts10@nhs.net (J.R.); zoe.adler@nhs.net (Z.A.); juliet.wright6@nhs.net (J.W.); 2 Department of Clinical and Experimental Medicine, Brighton and Sussex Medical School, Falmer, Brighton BN1 9PX, UK; 3 Department of Global Health and Infection, Brighton and Sussex Medical School, Falmer, Brighton BN1 9PX, UK; K.Alford2@bsms.ac.uk; 4 Department of Medical Education, Brighton and Sussex Medical School, Falmer, Brighton BN1 9PX, UK

**Keywords:** HIV, geriatrics, comprehensive geriatric assessment

## Abstract

As life expectancy in people living with HIV (PLWH) has increased, the focus of management has shifted to preventing and treating chronic illnesses, but few services exist for the assessment and management of these individuals. Here, we provide an initial description of a geriatric service for people living with HIV and present data from a service evaluation undertaken in the clinic. We conducted an evaluation of the first 52 patients seen in the clinic between 2016 and 2019. We present patient demographic data, assessment outcomes, diagnoses given, and interventions delivered to those seen in the clinic. The average age of attendees was 67. Primary reasons for referral to the clinic included management of complex comorbidities, polypharmacy, and suspected geriatric syndrome (falls, frailty, poor mobility, or cognitive decline). The median (range) number of comorbidities and comedications (non-antiretrovirals) was 7 (2–19) and 9 (1–15), respectively. All attendees had an undetectable viral load. Geriatric syndromes were observed in 26 (50%) patients reviewed in the clinic, with frailty and mental health disease being the most common syndromes. Interventions offered to patients included combination antiretroviral therapy modification, further health investigations, signposting to rehabilitation or social care services, and in-clinic advice. High levels of acceptability among patients and healthcare professionals were reported. The evaluation suggests that specialist geriatric HIV services might play a role in the management of older people with HIV with geriatric syndromes.

## 1. Introduction

Increased life expectancy in people living with HIV (PLWH) has brought the challenges of ageing and age-related issues to HIV clinical care [1]. In the UK, 39% of those accessing HIV services are now aged 50 and over, representing over 36,000 individuals considered “older” with HIV [2]. Cohort ageing is set to continue, with modelling work based on PLWH in the Netherlands predicting that, by 2030, 73% will be aged over 50 [3]. Importantly, as PLWH grow older, they appear to be experiencing disproportionally more age-related comorbidities than age-matched HIV-negative populations [4,5,6]. This is accompanied by greater polypharmacy, as well as issues of functional and cognitive decline, frailty, and falls [7,8]. These issues may be grouped as “geriatric syndromes”, highlighting a role for geriatric/elderly medicine within current HIV care [8,9,10].

How best to deliver geriatric care to PLWH remains unclear. Some service providers advocate for dedicated HIV-ageing services, and a small number of such services have developed or are developing around the world [11]. Joint HIV/speciality clinics have been effective within other specialties [12,13], therefore opening the door to the possibility of HIV-ageing clinics. However, outside of single-organ specialties, the clinical need and criteria for referral are harder to define. One approach is to clinically assess patients at a set age, such as 50 [14], though with the median age of HIV services users in the UK at 46, demand may be excessively high. An alternative could be the use of frailty or frailty syndromes in a “needs-based approach”. Tools to identify patients at risk of frailty using scoring methods are increasingly used internationally and have recently been integrated into UK primary care [15]. In October 2019, the European AIDS Clinical Society (EACS) published the first HIV guidance that advocates for frailty screening in older PLWH.

Frailty represents a reduction in physiological reserve that makes one vulnerable to adverse health outcomes [16]. It is prevalent in PLWH and has been associated with falls, incident multi-morbidity, hospitalisation, and death [17,18,19]. Frailty may present non-specifically (fatigue, weight loss) or as frailty syndromes such as falls, functional decline, and immobility. Additionally, frailty may contribute to medical complexity (polypharmacy and multi-morbidity) and has been associated with low mood and cognitive decline [20,21]. The recommended approach to the assessment and management of frailty is through Comprehensive Geriatric Assessment (CGA) [22]. Proactive identification of frailty and the introduction of CGA can enable early intervention to help PLWH to remain independent. CGA is a multidimensional, interdisciplinary diagnostic process used to determine the medical, psychosocial, and functional capabilities of older adults. Evidence suggests that CGA-based care can improve functional capacity and reduce the risk of institutionalisation when applied to other chronic conditions such as cancer, renal disease, and cardiovascular disease [23,24,25].

Older PLWH may face a complicated healthcare landscape [26], with HIV-specific management falling down the list of health priorities in favour of age-related issues, which HIV services may be ill-equipped to deal with due to lack of time, resources, or geriatric expertise.

In order to address the issues facing this ageing cohort, the Silver Clinic was established in the Brighton and Sussex University Hospitals Trust. This multi-disciplinary HIV-ageing clinic utilises a CGA approach to assess and manage age-related problems in PLWH. This paper aims to describe the service and results of an initial service evaluation of the clinic.

## 2. Methods

The Silver Clinic team consists of an (i) HIV consultant physician, (ii) Geriatrics consultant physician, (iii) HIV nurse specialist, and (iv) HIV pharmacist. The clinic operates monthly within the HIV outpatient (Lawson) unit. Clinic referrals come from any HIV healthcare professional (HCP) involved in the care of the patient. The Silver Clinic team is blinded to the process of referral. Current referral criteria are age (>50 years), presence of complex comorbidities and/or polypharmacy, or geriatric syndromes including frailty, falls, and difficulties with activities of daily living. All patients attending the clinic up until October 2019 were included in this evaluation. No exclusion criteria were applied. As this was a service evaluation, ethical approval was not obtained following assessment by the UK Health Research Authority (http://www.hra-decisiontools.org.uk/research/). Written inform consent was provided for the case study.

### 2.1. Clinic Process

**Figure 1:**
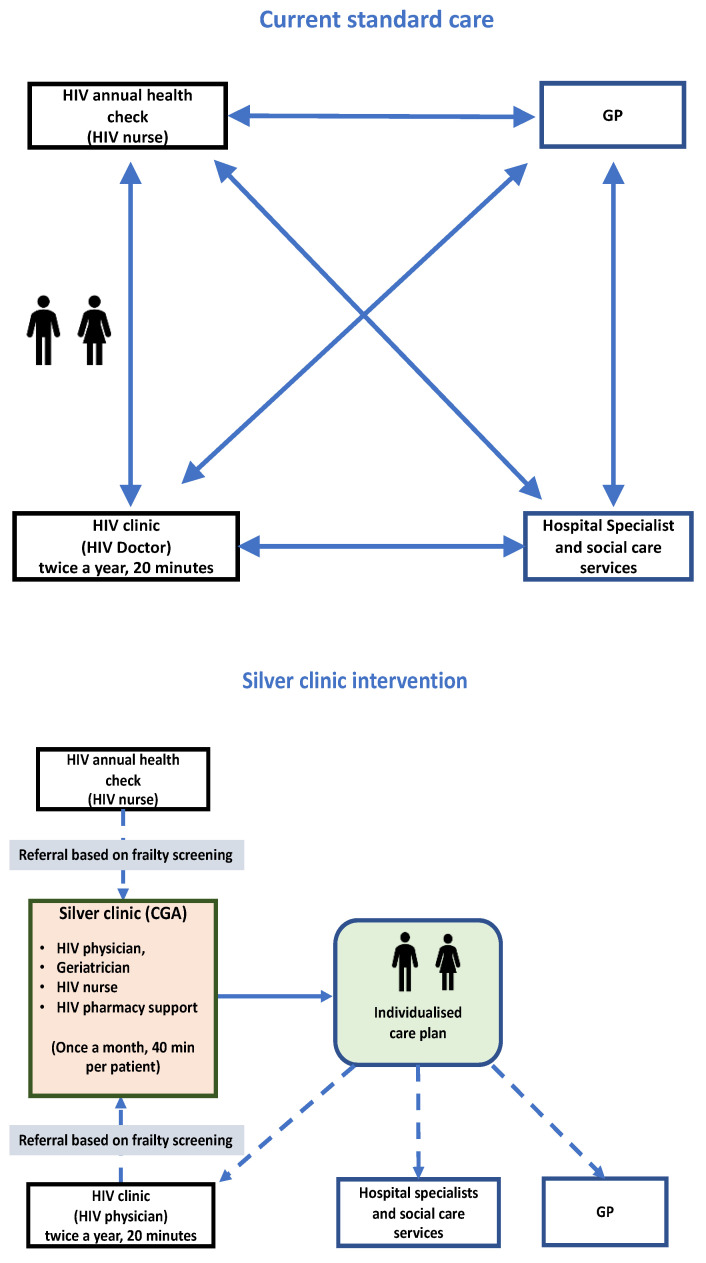
Silver Clinic process and position alongside standard care.

Before attending, new patients are asked to complete a number of screening questionnaires focussed around physical, functional, mood, and cognitive status. The questionnaires are patient-reported outcome measure (PROM) tools that serve two purposes. Firstly, they help practitioners to identify medical, social, or mental health issues that patients have before they are seen in the clinic. Secondly, they can be used to monitor the impact of the service. Prior to first clinic attendance, a multidisciplinary case-based discussion of each patient is organised. This includes: background and PROM review, evaluation of current clinical problems, and anticipated need of further investigations. All patients then receive a dual consultation with the HIV and elderly medicine physician focused on CGA. This explores patient demographics; social characteristics; comorbidity (including medications); functional, physical, mental health, and frailty status (Table 1). All assessments are triangulated by the multidisciplinary (MDT) to generate a comprehensive individualised management plan that is overseen by the Silver Clinic team and communicated to the referring clinician and the GP where the patient consents. The clinic process for the service is shown in Figure 1.

### 2.2. Clinical Assessments

Clinical data are drawn from patient notes and most recent routine HIV health checks. Baseline observations, including postural blood pressure and body mass index (BMI), are performed. Blood tests are taken to exclude issues contributing to age-related comorbidities, including vitamin B12 and folate deficiency, associated with neuropsychiatric issues, depression, and demyelinating myelopathy; calcium and 25-OH-Vitamin-D for bone health, falls, and mobility. The full assessment strategy is shown in Error! Reference source not found.

Mood symptoms are assessed using the Hospital Anxiety and Depression Scale (HADS)**,** a short, self-report screening questionnaire for generalised anxiety and depression among patients in non-psychiatric settings. The questionnaire is split into two sub-scales for anxiety (HADS-A) and depression (HADS-D), in which a score ≥11 is considered diagnostic [27].

Patient-reported outcome measures (PROM) include the Older Peoples’ Quality of Life Questionnaire (OPQL-brief), a 13-item validated tool for assessing quality of life (QoL) in older people. Scores range from 13 to 65, indicating lowest to highest QoL [28,29]. EuroQol five-dimension descriptive system (EQ-5D-5L) is a brief self-reported measure of generic health and perceived health status that has been used across a number of health conditions and populations [30,31]. The tool includes a visual analogue scale on which individuals indicate their current health state in relation to best and worst imaginable health (100–0, respectively). FRAIL Scale is a clinical screening tool for the identification of frailty. It comprises five self-reported components of fatigue—resistance, ambulation, illnesses, and weight loss—which are scored as present or absent, resulting in a score from 0 to 5. Those scoring 0 are robust, 1–2 prefrail and ≥3 frail [32].

Changes in scores for EQ-5D-5L and OPQL-brief and FRAIL scale at baseline (first assessment) and discharge (12 months) were calculated.

### 2.3. Patient and Healthcare Professional Satisfaction

A voluntary self-completed survey was completed by patients after the clinic appointment. The survey asked patients to provide qualitative “free-text” feedback of the service, including whether and how it benefitted them and suggestions for service improvement.

An 8-question online questionnaire for healthcare professionals, excluding those working in the Silver Clinic, was accessible from January to March 2018. This was created and hosted using Bristol Online Surveys and distributed via email to all HIV staff. Job role, clinic awareness and referral experience, perceived importance of the clinic, and improvement to older PLWH care were assessed. Partially completed questionnaires were omitted from analysis.

### 2.4. Statistical Analysis

Descriptive statistics using frequency, mean, or median with respective corresponding percentages, standard deviation, and interquartile range were used to summarize the data. Paired sample *t*-tests were used to evaluate the impact of the Silver Clinic intervention on scores for each PROM (EQ-5D-5L and OPQL-brief and FRAIL scale) from baseline to discharge from the clinic. A framework method of analysis was employed for qualitative data, with frequencies and percentages reported for qualitative variables where grouping was possible [33].

## 3. Results

From January 2015 to October 2019, the Silver clinic assessed 52 patients. Demographic characteristics, HIV, and other clinical data of clinic attendees are presented in Table 2. The median (range) age of attendees was 67 years (53–87), and the majority were white males identifying as men that have sex with men (MSM), reflecting the clinic population. Attendees had well-controlled chronic HIV, with 100% virally suppressed. Primary referral reason for patients attending the clinic was 67% (35) multimorbidity optimisation, 13% (7) problematic polypharmacy, and 17% (10) suspected geriatric syndrome (falls, frailty, mobility issues, cognitive decline).

**Figure 2:**
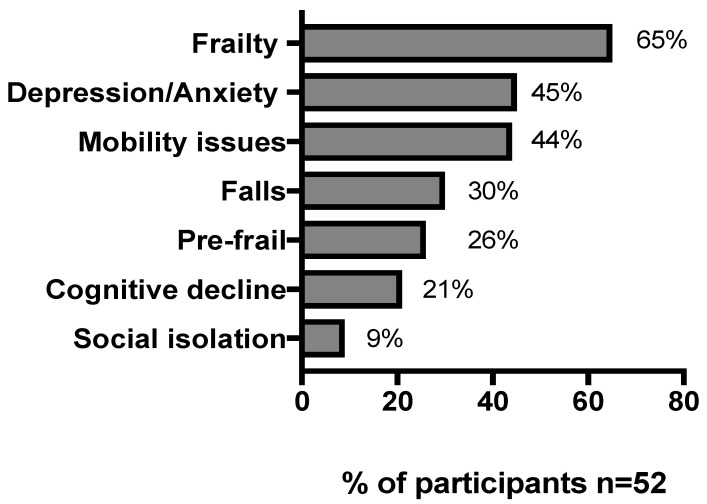
Frequencies of geriatric syndromes.

Geriatric syndromes were observed in all patients reviewed in the clinic, with frailty and mental health disease being the most common syndromes, as shown in Figure 2. Patients had a median (IQR) of 7 (2–19) comorbidities. Cardiovascular disease was the most common, reported by 36 patients (70%), followed by neurological disorder (63%), chronic pain syndrome (44%), and mental health conditions (42%). Polypharmacy defined as more than five non-antiretroviral drugs was common, with a median of nine (1–15) medications in addition to their ARVs. The most common comedications at first assessment were cardiovascular medications, followed by analgesics (61%), mental health drugs (40%), and supplements such as vitamins, etc. (53%). Notably, 30% were taking opioid medications for pain and 19% taking benzodiazepines to manage insomnia.

### 3.1. Patient-Reported Outcomes

Perceived health-related QoL at first assessment was poor on both the EQ-5D-5L and OPQOL, with mean scores (SD) for the visual scale on the EQ-5D-5L 56.76 (21.2) and 32.76 (8.48) for the OPQOL, respectively. Thirty-four (65%) patients were classified as frail; 14 (26%) were deemed prefrail and 4 (4%) robust using the FRAIL scale. Mental health was assessed using the HADS: 46% (23) had depression or anxiety symptoms at first assessment. No significant changes in EQ-5D-5L and OPQOL-Brief scores were observed at discharge compared to baseline (*p* = 0.885 and *p* = 0.218, respectively). Moreover, no changes in frailty status were observed at discharge using the FRAIL scale (*p* = 0.495)

### 3.2. Clinic Outcomes

At the point of data analysis, most patients had attended the clinic at least twice, with the median number of visits being two (2–8). The greatest number of visits was eight (two patients). ARV switch recommendations were made in six patients due to toxicities or drug–drug interactions (DDI). Twenty-seven specialist referrals were made, including to the broader multidisciplinary team including physiotherapy and occupational therapy. The clinic has now discharged 42 patients, 4 await follow-up appointment and 6 patients remain open to the service “as needed.

#### Case Study

To better understand that type of patients we see in the service we described the case of Mr X, a 69-year-old male who was diagnosed with HIV in 1999. He attended the Silver Clinic with complaints of feeling “fed up” and intermittent faecal incontinence. He has multiple comorbidities with associated polypharmacy, as shown in Table 3. He is an ex-smoker who drinks less than 4 units/week, with no recreational drug use. He lives alone and reports feeling socially isolated. He has poor mobility but dislikes using a recommended walking stick and reports three falls during the last 3 months. He relies heavily on his car as he lives in a rural village. His social benefits were reduced last year. His HIV is well controlled, with a current CD4 count of 750 cell/mL and undetectable viral load on a regimen of Darunavir, Ritonavir, and Lamivudine (3TC). His Q-Risk score was 16.5%.

1. Proactive treatment of constipation (causing overflow incontinence) and opioid reduction.
2. Fall prevention through occupational and physiotherapy referral and bone scan with subsequent osteoporosis treatment to reduce fracture risk.
3. Medicines rationalisation with ART modification to a one tablet regimen (Rezolsta: Darunavir/Cobistat), which in turn allowed Zopiclone withdrawal.
4. Signposting to community peer services aimed at increasing socialisation and an application for a disabled parking “Blue Badge” was supported.
5. Referral to Cognitive Behavioural Therapy with the hope that improvements in mental health may also stem from comorbidity optimisation.

At follow-up, despite subjectively his symptoms persisting, QOL based on the EQ-5D-5L and OPQOL had improved.

### 3.3. Patient Satisfaction

All patients were asked to provide their views of the clinic. Fourteen responses (35%) were received, with 13 (93%) stating that they were “very satisfied” with the service they received, and one respondent was “somewhat satisfied”. Qualitative feedback themes identified among respondents were “friendliness/kindness” (28%), “felt listened to” (14%), professional/helpful service (43%). Additional comments referred to the opportunity for in-depth explanation and care coordination.

### 3.4. Healthcare Professionals’ Views of the Silver Clinic

Fourteen (63%) staff responded to our invitation to provide views on the clinic; this included six (43%) doctors, six (43%) nurses, and two (14%) pharmacists. All participants were aware of the clinic, and six (42%) had made referrals. Twelve (85%) believed the Silver Clinic was “very important” for the management of older PLWH and the other two (14%) believed it was “quite important”. All respondents felt that the clinic improved the management of older PLWH.

## 4. Discussion

HIV services have a history of being proactive in their innovation of care models to address the changing needs of their patients. Clear trajectories towards HIV cohort ageing exists in the UK with accompanying age-related medical and psychosocial issues [34]. The Silver Clinic was created to address this emerging need—by employing multidisciplinary working and principles of geriatric care, it has sought to improve the care management of this complex cohort. This service evaluation demonstrates that the average age of Silver Clinic attendees was 67 years old, with the majority referred for management of multimorbidity, polypharmacy, and geriatric/frailty syndromes, which one might anticipate within general elderly medical services.

Classical geriatric syndromes have been demonstrated in older adults, with HIV at higher frequency and earlier age than one might expect for the general population. In one US study of 155 PLWH, median age 57, the prevalence of falls was 26%, and 54% described two or more geriatric syndromes, which were associated with greater comorbidity and lower nadir CD4 count [8]. In our clinic, cohort geriatric syndromes were common, with 44% complaining of mobility issues and 30% experiencing falls. These may be linked to other observed findings of mood disorder in 45% and social isolation in 9%. Social isolation was shown to be more common in older adults with HIV compared to those without (59% vs. 51%, *p* < 0.001) in the Veterans Aging Cohort Study and was associated with increased hospitalisation and mortality [35].

The majority of clinic attendees were frail (65%) based on the FRAIL scale, with a further 26% deemed prefrail. This is considerably higher than frailty prevalence seen in previous studies of PLWH, where frailty based on the Fried Phenotype (FP) ranged from 3 to 28% [36]; using this same measure, prevalence was 3% for frailty and 38% prefrailty in a large UK community study of adults aged 37–73 years [37]. The larger proportion with frailty in our group is reflective of the service model and its remit as a needs-based service, with frailty syndromes within the referral criteria. Targeting of frail and prefrail individuals is vital, however, as both are associated with an increased risk of incident disability in both personal and instrumental activities of daily living and mortality compared to robust individuals [32,38]. Though the natural trajectory of frailty is progressive, studies have demonstrated stability in frail state at one year, as was demonstrated in this non-interventional evaluation of the service [39]. The ongoing presence of frailty in this selected cohort suggests that they may have the most to gain from ongoing geriatric medical input.

The clinic users had a high burden of comorbidity and polypharmacy, particularly mood disorder (45%) and chronic pain, with 30% on regular opiates. High levels of comorbidity and polypharmacy have been noted in cohorts of PLWH [14,40], with both related to greater duration of HIV rather than older age [40]. Chronic opiate use for non-cancer pain is higher in PLWH and is associated with greater comorbidity, and it is an independent risk factor for falls, along with other medications such as benzodiazepines, in this group [41,42]. Comorbidity and polypharmacy, including chronic pain, combined with both frailty and broader geriatric syndromes, would support a multimorbidity-based approach as advocated by the National Institute of Health and Care Excellence. The use of patient-centred care focused around how one’s comorbidities and their treatments impact on quality of life and how they align with their life priorities, emphasising strong care coordination [43]. These models are compatible with geriatric principles of care that have been advocated within the HIV literature [11,44,45]. Patient-reported QoL was poor at baseline, with no significant change at one year. This likely reflects the level of comorbidity and functional limitation, alongside unmeasured psychosocial factors [46]. Whilst the former were addressed from a diagnostic perspective, service users could benefit from broader MDT intervention, care coordination, and community-based intervention centred on social prescribing and peer support [47].

It was gratifying to see that the survey of HCPs indicated appropriate awareness of the Silver Clinic, with many reporting the positive improvement to the management of older PLWH as a consequence of the clinic. However, a recent UK survey identified the existence of only two dedicated HIV-ageing services nationwide, with two-thirds of respondents citing insufficient population as the reason for no perceived need for such a service [48], yet we know that a predominantly older adult HIV population is predicted in the near future [3]. HIV-ageing services are now being reported more widely, mainly in high income settings, which are either based on geriatric syndromes or metabolic comorbidities in PLWH [49]. Attendees reported high levels of satisfaction with the clinic, though it should be noted that the number providing feedback was self-selecting and small (35%). However, service user feedback is vital in both the development and evaluation of any new ageing services, with respondents in one survey favouring the maintenance of care within HIV settings, alongside enhanced communication and care coordination [50].

To our knowledge, this represents the first evaluation of a UK-based joint HIV-ageing service run on a needs- rather than age-based approach that utilises CGA principles. It is limited in its presentation of single-site data that are purely descriptive in nature. Other limitations include the small sample size, which also limits the generalizability and reproducibility of the data. Data presented here are not able to define the longer-term benefit regarding preservation of function, admission avoidance, and mortality, as well as the cost-effectiveness of attending the Silver Clinic. However, this is the case for many new services, which are not, at present, being driven by a supporting evidence base. The role of such a clinic, and in particular the role of CGA applied to individuals living with HIV, represents areas of research need within HIV and ageing, which is supported by a nationwide service evaluation on HIV-ageing services that identified a lack of evidence base as a barrier for service development [48]. However, the Silver Clinic is a trail-blazer for other services, with a model that, since conception, meets the recommendations from both the 2019 EACS guidance on frailty screening in PLWH [51] and the 2018 British HIV Association Standards of Care document, which advocates the involvement of a geriatrician with HIV knowledge in the care of service users requiring complex HIV care [52].

## 5. Conclusions

We have operationalised a dedicated HIV-ageing service founded on geriatric medicine principles that is acceptable to both service users and referrers within HIV medicine. Referral pathways have identified a clinic cohort with high burden of frailty, comorbidity, and geriatric syndromes that might benefit from comprehensive geriatric assessment. Ongoing evaluation and research in this area is crucial to demonstrate the effectiveness of this model of care and/or help in building an evidence base to support other models of care for those ageing with HIV.

## Figures and Tables

**Table 1:** Clinical assessments performed within the Silver Clinic.

Test Category	
**Blood Tests**	Calcium, TFTs, PSA, HbA1c, B12/Folate, Vitamin D
**HIV clinical data**	Year of diagnosis, nadir and current CD4 cell count, current CD8 cell count, CD4:CD8 ratio, antiretroviral history
**Other clinical data**	Urinalysis, height, weight, body mass index, blood pressure (lying and standing)
**Mood assessment**	Hospital Anxiety and Depression Scale
**Frailty assessment**	FRAIL scale
**Patient reported outcome measures**	Euroqol-5D-5L Older Peoples’ Quality of Life Questionnaire

TFTs, thyroid function tests; PSA, prostate-specific antigens; HbA1c, Haemoglobin A1c.

**Table 2:** Summary of patient characteristics.

Clinical Characteristics (*n* = 52)	Median (IQR) Otherwise Stated
Age (years)	67 (53–87)
Male, *n* (%)	47 (90)
White ethnicity, *n* (%)	50 (96)
Identified sexuality, *n* (%)	
MSM	41 (78)
Heterosexual	9 (17)
Other	2 (4)
Current smoker, *n* (%)	17 (32)
Alcohol use, *n* (%)	
Less than 10 units per week	41(77)
Between 10 and 20 units per week	5 (9)
>20 units per week	3 (4)
Recreational drug use *n* (%)	6 (11)
Comorbidities	7 (2–19)
Comedications	9 (1–15)
QRISK3 *	25 (6–52)
Bone densitometry, *n* (%)	
Osteoporosis	15 (28)
Osteopenia	33 (63)
Normal BMD	4 (7)
**HIV Clinical Parameters**	
Time since HIV diagnosis: years (median; range)	17 (6–34)
Duration of cART: years (median; range)	17 (5–30)
cART-based regimen *n* (%)	
Protease inhibitor	28 (53)
NNRTI	12 (23)
INSTI	12 (23)
HIV RNA < 50 copies/mL, *n* (%)	52 (100)
Nadir CD4 (cells/μL)	287 (223)
Current CD4 (cells/μL)	563 (368)
CD4:CD8 ratio	0.60 (0.1)

MSM, men who have sex with men; cART, antiretroviral therapy; QRISK^®^3-2018 cardiovascular risk calculator.

**Table 3:** Mr X comorbidities and medications.

Medical Comorbidities	Comedications
Type 2 diabetes Depression Osteopenia Dyslipidaemia Chronic back pain secondary to (degenerative disease and C-spine injury) Peripheral neuropathy (ART-related) Chronic inflammatory demyelinating polyneuropathy unresponsive to immunoglobulin	1. Paroxetine 40 mg daily 2. Gabapentin 400 mg twice daily 3. Aspirin 75 mg daily 4. Zopiclone 7.5 mg at night 5. Pravastatin 10 mg at night 6. Zomorph MR 60 mg twice daily 7. Metformin 1 g twice daily 8. Folic Acid 5 mg daily 9. Oramorph 5−10 mg as needed

## References

[1] Harris T.G., Rabkin M., El-Sadr W.M. (2018). Achieving the fourth 90. AIDS.

[2] Nash S., Desai S., Croxford S., Guerra L., Lowndes C., Connor N., Gill O.N. (2018). Progress towards Ending the HIV Epidemic in the United Kingdom: 2018 Report.

[3] Smit M., Brinkman K., Geerlings S., Smit C., Thyagarajan K., Van Sighem A., De Wolf F., Hallett T.B. (2015). Future challenges for clinical care of an ageing population infected with HIV: A modelling study. Lancet Infect. Dis..

[4] Schouten J., Wit F.W., Stolte I.G., Kootstra N.A., Van Der Valk M., Geerlings S.E., Prins M., Reiss P., Kooij K.W., Van Zoest R.A. (2014). Cross-sectional comparison of the prevalence of age-associated comorbidities and their risk factors between HIV-infected and uninfected individuals: The AGEhIV cohort study. Clin. Infect. Dis..

[5] Althoff K.N., Jacobson L.P., Cranston R.D., Detels R., Phair J.P., Li X., Margolick J.B., for the Multicenter AIDS cohort study (MACS) (2013). Age, comorbidities, and AIDS predict a frailty phenotype in men who have sex with men. J. Gerontol. Ser. A Biol. Sci. Med. Sci..

[6] Guaraldi G., Orlando G., Zona S., Menozzi M., Carli F., Garlassi E., Berti A., Rossi E., Roverato A., Palella F. (2011). Premature age-related comorbidities among HIV-infected persons compared with the general population. Clin. Infect. Dis..

[7] Halloran M., Boyle C., Kehoe B., Bagkeris E., Mallon P., A Post F., Vera J., Williams I., Anderson J., Winston A. (2019). Polypharmacy and drug–drug interactions in older and younger people living with HIV: The POPPY study. Antivir. Ther..

[8] Greene M., Covinsky K.E., Valcour V., Miao Y., Madamba J., Lampiris H., Cenzer I.S., Martin J., Deeks S.G. (2015). Geriatric syndromes in older HIV-infected adults. J. Acquir. Immune Defic. Syndr..

[9] Guaraldi G., Rockwood K. (2017). Geriatric-HIV medicine is born. Clin. Infect. Dis..

[10] Hawkins K.L., Brown T.T., Margolick J.B., Erlandson K.M. (2017). Geriatric syndromes: New frontiers in HIV and sarcopenia. AIDS.

[11] Singh H.K., Del Carmen T., Freeman R., Glesby M.J., Siegler E.L. (2017). From one syndrome to many: Incorporating geriatric consultation into HIV care. Clin. Infect. Dis..

[12] McClure M., Singh G.J., Rayment M., Jones R., Levy J.B. (2012). Clinical outcomes of a combined HIV and renal clinic. Clin. Kidney J..

[13] Koganti S., Loes S.K.-D., Hutchinson S., Johnson M., Rakhit R.D. (2015). Management of cardiovascular conditions in a cohort of patients with HIV: Experience from a joint HIV/cardiology clinic. Clin. Med..

[14] Waters L., Patterson B., Scourfield A., Hughes A., De Silva S., Gazzard B., Barton S., Asboe D., Pozniak A., Boffito M. (2012). A dedicated clinic for HIV-positive individuals over 50 years of age: A multidisciplinary experience. Int. J. STD AIDS.

[15] NHS England, LTC Team (2017). Toolkit for General Practice in Supporting Older People Living with Frailty.

[16] Clegg A., Young J., Iliffe S., Rikkert M.O., Rockwood K. (2013). Frailty in elderly people. Lancet.

[17] Tassiopoulos K., Abdo M., Wu K., Koletar S.L., Palella F.J., Kalayjian R., Taiwo B., Erlandson K.M. (2017). Frailty is strongly associated with increased risk of recurrent falls among older HIV-infected adults. AIDS.

[18] Guaraldi G., Brothers T.D., Zona S., Stentarelli C., Carli F., Malagoli A., Santoro A., Menozzi M., Mussi C., Mussini C. (2015). A frailty index predicts survival and incident multimorbidity independent of markers of HIV disease severity. AIDS.

[19] Akgun K.M., Tate J.P., Crothers K., Crystal S., Leaf D.A., Womack J., Brown T.T., Justice A.C., Oursler K.K. (2014). An adapted frailty-related phenotype and the VACS index as predictors of hospitalization and mortality in HIV-infected and uninfected individuals. J. Acquir. Immune Defic. Syndr..

[20] Collard R.M., Comijs H.C., Naarding P., Penninx B.W., Milaneschi Y., Ferrucci L., Voshaar R.C.O. (2015). Frailty as a predictor of the incidence and course of depressed mood. J. Am. Med. Dir. Assoc..

[21] Underwood J., Robertson K.R., Winston A. (2015). Could antiretroviral neurotoxicity play a role in the pathogenesis of cognitive impairment in treated HIV disease?. AIDS.

[22] (2017). British Geriatric Society, Fit for Frailty. https://www.bgs.org.uk/sites/default/files/content/resources/files/2018-05-14/fff2_short.pdf.

[23] Ellis G., Gardner M., Tsiachristas A., Langhorne P., Burke O., Harwood R.H., Conroy S.P., Kircher T., Somme D., Saltvedt I. Comprehensive geriatric assessment for older adults admitted to hospital. Cochrane Database Syst. Rev..

[24] Stuck A.E., Iliffe S. (2011). Comprehensive geriatric assessment for older adults. BMJ.

[25] Garrard J.W., Cox N.J., Dodds R.M., Roberts H.C., Sayer A.A. (2019). Comprehensive geriatric assessment in primary care: A systematic review. Aging Clin. Exp. Res..

[26] (2017). Terrence Higgins Trust, Uncharted Territory: A Report into the First Generation Growing Older with HIV. https://www.tht.org.uk/sites/default/files/2018-03/uncharted_territory_final_low-res.pdf.

[27] Zigmond A.S., Snaith R.P. (1983). The hospital anxiety and depression scale. Acta Psychiatr. Scand..

[28] Bowling A., Hankins M., Windle G., Bilotta C., Grant R. (2013). A short measure of quality of life in older age: The performance of the brief older people’s quality of life questionnaire (OPQOL-brief). Arch. Gerontol. Geriatr..

[29] Kaambwa B., Gill L., McCaffrey N., Lancsar E., Cameron I.D., Crotty M., Gray L., Ratcliffe J. (2015). An empirical comparison of the OPQoL-Brief, EQ-5D-3 L and ASCOT in a community dwelling population of older people. Health Qual. Life Outcomes.

[30] Herdman M., Gudex C., Lloyd A., Janssen M., Kind P., Parkin D., Bonsel G., Badia X. (2011). Development and preliminary testing of the new five-level version of EQ-5D (EQ-5D-5L). Qual. Life Res..

[31] Dyer M.T.D., A Goldsmith K., Sharples L.S., Buxton M.J. (2010). A review of health utilities using the EQ-5D in studies of cardiovascular disease. Health Qual. Life Outcomes.

[32] Kojima G. (2018). Quick and Simple FRAIL Scale predicts incident activities of daily living (ADL) and instrumental ADL (IADL) disabilities: A systematic review and meta-analysis. J. Am. Med. Dir. Assoc..

[33] Gale N.K., Heath G., Cameron E., Rashid S., Redwood S. (2013). Using the framework method for the analysis of qualitative data in multi-disciplinary health research. BMC Med. Res. Methodol..

[34] Bagkeris E., Burgess L., Mallon P.W., Post F.A., Boffito M., Sachikonye M., Anderson J., Asboe D., Garvey L., Vera J. (2018). Cohort profile: The Pharmacokinetic and clinical observations in PeoPle over fiftY (POPPY) study. Int. J. Epidemiol..

[35] Greysen S.R., Horwitz L.I., Covinsky K.E., Gordon K., Ohl M., Justice A.C. (2013). Does social isolation predict hospitalization and mortality among HIV+ and uninfected older veterans?. J. Am. Geriatr. Soc..

[36] Levett T.J., Cresswell F.V., Malik M.A., Fisher M., Wright J. (2016). Systematic Review of Prevalence and predictors of frailty in individuals with human immunodeficiency virus. J. Am. Geriatr. Soc..

[37] Hanlon P., I Nicholl B., Jani B.D., Lee D., McQueenie R., Mair F.S. (2018). Frailty and pre-frailty in middle-aged and older adults and its association with multimorbidity and mortality: A prospective analysis of 493 737 UK Biobank participants. Lancet Public Health.

[38] Kojima G. (2018). Frailty Defined by FRAIL Scale as a predictor of mortality: A systematic review and meta-analysis. J. Am. Med. Dir. Assoc..

[39] Gill T.M., Gahbauer E.A., Allore H.G., Han L. (2006). Transitions between frailty states among community-living older persons. Arch. Intern. Med..

[40] Guaraldi G., Malagoli A., Calcagno A., Mussi C., Celesia B.M., Carli F., Piconi S., De Socio G.V., Cattelan A.M., Orofino G. (2018). The increasing burden and complexity of multi-morbidity and polypharmacy in geriatric HIV patients: A cross sectional study of people aged 65–74 years and more than 75 years. BMC Geriatr..

[41] Silverberg M.J., Ray G.T., Saunders K., Rutter C.M., Campbell C.I., Merrill J.O., Sullivan M.D., Banta-Green C.J., Von Korff M., Weisner C. (2012). Prescription long-term opioid use in HIV-infected patients. Clin. J. Pain.

[42] Erlandson K.M., Allshouse A.A., Jankowski C.M., Duong S., Mawhinney S., Kohrt W.M., Campbell T.B. (2012). Risk factors for falls in HIV-infected persons. J. Acquir. Immune Defic. Syndr..

[43] (2016). NICE, Multimorbidity: Clinical Assessment and Management. National Institute of Health Research Guidelines. https://www.nice.org.uk/guidance/ng56.

[44] Greene M., Justice A.C., Covinsky K.E. (2016). Assessment of geriatric syndromes and physical function in people living with HIV. Virulence.

[45] Levett T., Wright J. (2017). How to assess and manage frailty in patients with HIV. Sex. Transm. Infect..

[46] Langebeek N., Kooij K.W., Wit F.W., Stolte I.G., Sprangers M.A.G., Reiss P., Nieuwkerk P.T. (2017). Impact of comorbidity and ageing on health-related quality of life in HIV-positive and HIV-negative individuals. AIDS.

[47] Vargas R.B., Cunningham W.E. (2006). Evolving trends in medical care-coordination for patients with HIV and AIDS. Curr. HIV/AIDS Rep..

[48] Cresswell F., Levett T. (2017). Specialist care of older adults with HIV infection in the UK: A service evaluation. HIV Med..

[49] Siegler E.L., O Burchett C., Glesby M.J. (2018). Older people with HIV are an essential part of the continuum of HIV care. J. Int. AIDS Soc..

[50] Pollard A., Llewellyn C., Cooper V., Sachikonye M., Perry N., Nixon E., Miners A., Youssef E., Sabin C. (2017). Patients’ perspectives on the development of HIV services to accommodate ageing with HIV: A qualitative study. Int. J. STD AIDS.

[51] European AIDS Clinical Society EACS. https://www.eacsociety.org/files/2019_guidelines-10.0_final.pdf.

[52] (2018). BHIVA, Standards of Care for People Living with HIV. https://www.bhiva.org/file/KrfaFqLZRlBhg/BHIVA-Standards-of-Care-2018.pdf.

